# Cancer mortality in workers at risk of occupational exposure to ionizing radiation in a company in the nuclear sector headquarters in São Paulo

**DOI:** 10.1590/1980-549720240011

**Published:** 2024-03-18

**Authors:** Glacy Sabra Vieira, Maria Carmen Martinez, Maria Regina Alves Cardoso

**Affiliations:** I Nuclear and Energy Research Institute – São Paulo (SP), Brazil.; II WAF Informática & Saúde Ltda – São Paulo (SP), Brazil.; III Universidade de São Paulo, School of Public Health of São Paulo – São Paulo (SP), Brazil.

**Keywords:** Ionizing radiation, Worker, Occupational exposure, Neoplasms., Mortality, Cohort studies, Radiação ionizante, Trabalhador, Exposição ocupacional, Neoplasias, Mortalidade, Estudos de coortes

## Abstract

**Objective:**

To compare cancer mortality among workers exposed to gamma and X radiation and the general population of the city of São Paulo, as well as that of the subgroup monitored with those not monitored for gamma and X radiation in a work unit with ionizing radiation based in the city of São Paulo.

**Methods:**

Between 2016 and 2021, a retrospective open cohort study was carried out with workers who were employed from 08/31/1956 to 12/31/2016 based on data collected at the company and in official institutions. Standardized mortality ratios (SMR) were calculated by sex, age and calendar period of cancers grouped according to type, risk factor and organ system in two analyses: in the external analysis, the mortality of the study population was compared with that of the general population of the city of São Paulo; In the internal analysis, the mortality of the monitored subgroup was compared with that of the subgroup not monitored for gamma and X radiation.

**Results:**

The external mortality analysis showed SMR=0.224 (95%CI 0.208–0.240) and the healthy worker effect, while the internal mortality analysis showed SMR=0.685 (95%CI 0.618–0.758).

**Conclusion:**

This study showed lower cancer mortality among exposed workers when compared to mortality in the general population and the healthy worker effect. Among workers monitored for gamma and X radiation, cancer mortality was lower when compared to those not monitored.

## RESUMO

**Objetivo:**

Comparar a mortalidade por câncer entre trabalhadores expostos à radiação gama e X e a população geral do município de São Paulo, bem como a do subgrupo monitorado com o não monitorado para radiação gama e X em uma unidade de trabalho sediada no município de São Paulo.

**Métodos:**

Entre 2016 e 2021 foi realizado estudo de coorte aberta retrospectiva com trabalhadores que tiveram vínculo empregatício desde 31/08/1956 até 31/12/2016 a partir de dados coletados na empresa e em instituições oficiais. Foram calculadas as razões de mortalidade padronizadas (RMP) por sexo, idade e período calendário de cânceres agrupados segundo o tipo, o fator de risco e o sistema orgânico em duas análises: na análise externa, comparou-se a mortalidade da população de estudo com a da população geral do município de São Paulo; já na análise interna, comparou-se a mortalidade do subgrupo monitorado com a do subgrupo não monitorado para radiação gama e X.

**Resultados:**

A análise externa de mortalidade mostrou RMP=0,224 (IC95% 0,208–0,240) e o efeito do trabalhador sadio, enquanto a análise interna de mortalidade mostrou RMP = 0,685 (IC95% 0,618–0,758).

**Conclusão:**

Este estudo mostrou menor mortalidade por câncer entre os trabalhadores expostos quando comparada com a mortalidade da população geral e o efeito do trabalhador sadio. Entre os trabalhadores monitorados para radiação gama e X, a mortalidade por câncer foi menor quando comparada com a dos não monitorados.

## INTRODUCTION

Ionizing radiation is a carcinogen, and the relationship between exposure dose and cancer risk is linear and limitless^1^. Safety limits for work are based on populations exposed to single high dose or on patients exposed to high doses of radiotherapy^2–4^, in addition to experimental studies; but occupational exposure is chronic at low doses. To clarify doubts about the validity of safety limits for work, cohort studies of nuclear industry workers were carried out in developed countries and did not demonstrate conclusive results regarding the association between occupational exposure to ionizing radiation and cancer mortality, although some positive associations have been found^1^ in relation to lung^5,6^ and prostate^7,8^ cancer, multiple myeloma^9–12^, and leukemia^10–14^. No studies of this nature were identified with Brazilian workers.

The questioning regarding illness and death from cancer resulting from exposure to ionizing radiation in the work environment from a radiological and nuclear company in the city of São Paulo, combined with the lack of national studies, gave rise to this study, which aimed to compare cancer mortality among workers exposed to ionizing radiation with mortality in the general population of the city of São Paulo and the subgroups monitored and not monitored for gamma and X radiation in a work unit based in the city of São Paulo.

## METHODS

### Study design and population

Open historical cohort in a target population of 6,394 workers from a public company in the city of São Paulo (SP) in the research, development, and applications in the radiological and nuclear areas. The cohort began on 08/31/1956, the date of the company's founding, and ended on 12/31/2016. Each worker was included in the cohort from the date they joined the company, and the end of follow-up corresponded to the date of death or the end of the cohort.

Inclusion criteria included having a formal employment record and at least one day of work at the company, after which workers would be at risk of exposure to gamma and X radiation. Workers who refused to participate were considered losses of the study as well as the lack of information about whether or not workers had passed away.

### Sources and data collection

Identification, demographic, and functional data were obtained from records from the company's administrative sector, that is: name, date of birth, affiliation, hometown, General Registration (Brazilian identification document – RG) of the Public Security Secretariats, Individual Taxpayer Registration (CPF), voter ID, start and end date of employment, initial education, positions occupied and date of death (if applicable). Information on exposure to gamma and X radiation was obtained from monthly dosimetric records from the reading of individual external chest dosimeters at the company from 1961 to 2016.

Mortality data were only available at the company and in official sources limited to the state of São Paulo, namely the Mortality Information Improvement Program of the Municipal Health Secretariat of the Municipality of São Paulo (*Programa de Aprimoramento de Informações de Mortalidade da Secretaria Municipal de Saúde do Município de São Paulo* – PRO-AIM), the Information Center Health Surveillance Strategies of the Health Secretariat of the state of São Paulo (*Centro de Informações Estratégicas de Vigilância em Saúde da Secretaria da Saúde do Estado de São Paulo* – CIEVS-SP), and the SEADE Foundation. Consultation of the databases in PRO-AIM and CIEVS-SP used the probabilistic linkage technique (by crossing the name, name of the mother, and date of birth) and in SEADE, deterministic linkage (by crossing the name, name of the mother, date of birth, name of the father, hometown, CPF, RG, dates of start and end of employment) with preference given to PRO-AIM records. The underlying cause of death was recorded according to the International Classification of Diseases – 10^th^ Revision (ICD-10).

To classify the vital status of each worker, workers reportedly dead were considered "deceased" and a worker who was receiving a salary or pension was considered "alive." By consulting the database and the homepage of the Regional Electoral Court with national coverage^15^ and the homepage of the Federal Revenue Service^15^, those with regular voter ID and CPF were considered "alive," those with death information were considered "deceased," and those missing information were considered "ignored."

Demographic and mortality records for the city of São Paulo were obtained from DATASUS^16^ from 1996 to 2016, and underlying causes of death were recorded according to ICD-10.

### Study variables

Sociodemographic and functional variables: gender, date of birth, start and end date of employment, end date of monitoring in the cohort, age at start and end of employment, age at death (deceased), age at end date of cohort (living), positions (categorized according to level of education into mid-level positions, higher level positions, transition from mid-level to higher level positions, and unknown).

Exposure to gamma and X radiation: were categorized into monitored and unmonitored subgroups for exposure to gamma and X radiation^17^.

Vital status: alive, deceased or unknown.

Causes of death: death records were categorized into all causes, all cancers, all causes except cancer, and by cancer site. Cancer categories considered, according to ICD-10, the group of malignant neoplasms (C00 to C97)^18^, *in situ* neoplasms (D00 – D09), benign neoplasm of brain^19^ (D32 and D33), and neoplasms of uncertain or unknown behavior (D37–D48). Deaths of unknown underlying cause were coded as R99 by the researcher.

Neoplasms were grouped according to type (solid, unspecified, and hematopoietic), risk factors (alcohol consumption, smoking, work, and exposure to gamma and X radiation)^20^, and organic systems according to the ICD-10 classification of neoplasms^16^ (Chart A of the Supplementary Material).

### Statistical analyses

Descriptive analysis stratified by gender of the quantitative variables was carried out using means, medians, standard deviations, minimum and maximum values; proportions were set from the qualitative variables. Crude mortality rates (CMR) were estimated by dividing the number of deaths observed by the number of person-years, which comprises the number of years that workers attended follow-up between the start and end dates from the cohort.

Compare the risk of mortality between populations (exposed and unexposed) was carried out by the use of standardized mortality ratio (SMR), obtained by dividing the number of deaths observed by the number of deaths expected using the indirect method^21,22^. SMR were stratified by gender, age, and calendar period (calculation method described in the Supplementary Material). A 95% confidence interval (95%CI) was considered for statistical significance.

External analysis compared mortality in the study population with that in the city of São Paulo from 1996 to 2016. Age groups at the end of follow-up were 15 to 34.9 years old, 35 to 49.9 years old, and 50 years old or older. Calendar periods were 01/01/1996–12/31/2002, 01/01/2003–12/31/2009, and 01/01/2010–12/31/2016. Internal analysis compared the mortality of monitored workers with the mortality of unmonitored workers from 1993 to 2016, given the reduced number of death records prior to 1993. Age groups at the end of follow-up were 15 to 34.9 years old, 35 to 49.9 years old, and 50 years old and older. Calendar periods were 01/01/1993–12/31/2000, 01/01/2001–12/31/2008, and 01/01/2009–12/31/2016.

### Software

Microsoft Excel, version 365, was used to calculate the crude and standardized rates. STATA – Statistics/Data Analysis, version 13.1, was used for descriptive analyses and the calculation of standardized ratios.

### Ethical aspects

The study was approved by the Research Ethics Committee of the School of Public Health of *Universidade de São Paulo*, CAAE No. 54944616.6.0000.5421 of 08/29/2016. The company authorized the study to be carried out with workers who signed the Informed Consent. For workers who could not be contacted, the Term of Responsibility was approved by the Research Ethics Committee of the Municipal Health Secretariat of the Municipality of São Paulo on 08/31/2018.

## RESULTS

**Figure 1:**
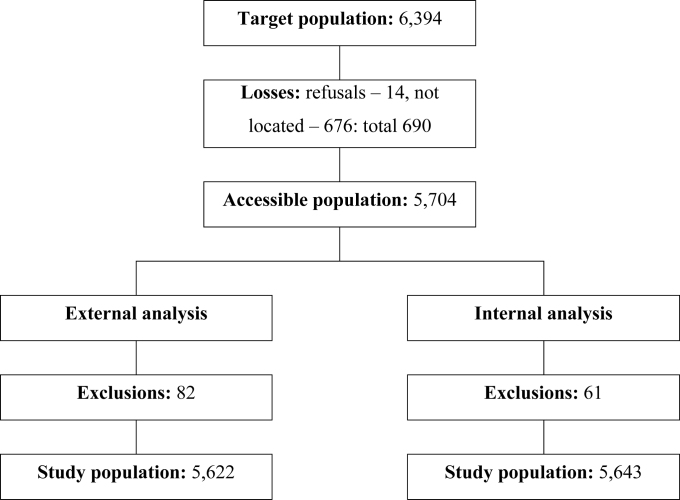
Target population and study population; company working in research, development, and applications in the radiological and nuclear areas, São Paulo, 1993–2016.

6,394 workers’ records were found, of which 676 were lost to follow-up and 14 refused to participate, totaling 690 (10.8%) losses, with 5,704 (89.2%) remaining workers. 82 workers who ended follow-up before 1996 were excluded from external analysis, leaving a study population of 5,622 (87.9%) workers. From internal analysis, 61 participants who ended follow-up before 1993 were excluded, making up a study population of 5,643 (88.3%) workers. Steps are illustrated in Figure 1.

Table 1 shows the characteristics of the study population, consisted majorly of males (74.0%), aged 60 years old or older (56.4%), with a mean of 61.7 years (SD=9.9).

**Table 1:** Characteristics of the study population according to gender; company working in research, development, and applications in the radiological and nuclear areas, São Paulo, 1993–2016.

Characteristics		Males		Females		Total	
		N°	%	N°	%	N°	%
Age range (in years) at the end of follow-up							
	15 ├ 35	42	1.0	15	1.0	57	1.0
	35 ├ 50	256	6.1	133	9.1	389	6.9
	50 ├ 60	1,542	36.9	473	32.2	2,015	35.7
	≥60	2,334	55.9	848	57.7	3,182	56.4
Calendar period for employment							
	1956 to 1959	45	1.1	9	0.6	54	1.0
	1960 to 1969	274	6.6	106	7.2	380	6.7
	1970 to 1979	1,810	43.4	727	49.5	2,537	45.0
	1980 to 1989	1,857	44.5	488	33.2	2,345	41.6
	1990 to 1999	98	2.3	84	5.7	182	3.2
	2000 to 2016	90	2.2	55	3.7	145	2.6
Length of employment (in years)							
	Less than 1.0	930	22.3	255	17.4	1,185	21.0
	1.0 to 4.9	1,621	38.8	457	31.1	2,078	36.8
	5.0 to 9.9	446	10.7	181	12.3	627	11.1
	10.0 to 19.9	342	8.2	163	11.1	505	8.9
	20.0 to 29.9	291	7.0	204	13.9	495	8.8
	30.0 to 39.9	473	11.3	177	12.0	650	11.5
	40.0 to 49.9	69	1.7	32	2.2	101	1.8
	50.0 or more	2	0.0	0	0.0	2	0.0
Characteristics of the position held							
	High-level position	273	6.5	183	12.5	456	8.1
	Mid-level position	2,046	49.0	774	52.7	2,820	50.0
	Hybrid position (mid- and higher-level)	1,789	42.9	443	30.2	2,232	39.6
	Unknown	66	1.6	69	4.7	135	2.4
Risk of exposure to gamma and X radiation							
	No	1,664	39.9	691	47.0	2,355	41.7
	Yes	2,510	60.1	778	53.0	3,288	58.3
Vital status at the end of follow-up							
	Alive	3,532	84.6	1,330	90.5	4,862	86.2
	Died from cancer	139	3.3	57	3.9	196	3.5
	Died from other causes	503	12.1	82	5.6	585	10.4
Total		4,174	100.0	1,469	100.0	5,643	100.0

For both genders, mean follow-up time was 35.3 years (SD=8.9). The majority of the population had been employed for less than 5 years (57.8%), with a mean of 9.5 years (SD=12.4).

Workers in mid-level positions were the most frequent (50.0%), followed by those who moved from mid- to higher level positions (39.6%).

External monitoring due to risk of exposure to gamma and X radiation was observed in 58.3% of workers. Among the workers monitored, 69.5% had an accumulated radiation dose below 10.0 mSv. The mean accumulated dose in the monitored subgroup was 14.9 mSv (SD=35.7), with a median of 3.0 mSv, a minimum of 0.0 mSV, and a maximum of 656.2 mSv.

At the end of follow-up, 781 (13.8%) workers had died. Cardiovascular diseases were the main causes of deaths, accounting for 224 of them (28.8%); neoplasms were the second most common cause of death, with 196 (25.1%) cases; and undefined or unspecified causes were the third, with 15.4% of deaths (the Supplementary Material presents Table A, which shows deaths according to ICD-10 chapters, and Table B, which shows deaths due to cancer by site).

In relation to the positions held, a higher proportion of cancer deaths was observed in the category of higher-level positions (38.0%), followed by the category of mid-level positions that migrated to higher-level ones (28.9%), higher-level positions (22.2%), and unknown (12.5%).

CMR for all causes was 629.2/100,000 person-year, being higher among men than women. CMR for all cancers was 157.9/100,000 person-year, being higher among women than men. Considering organic systems, the largest CMR due to neoplasms were in the digestive (64.4/100,000), respiratory (33.8/100,000), and genitourinary systems (29.0/100,000), and hematogenous neoplasms (14.5/100,000) (Table 2).

**Table 2:** Distribution of deaths according to cause and gender; company working in research, development, and applications in the radiological and nuclear areas, São Paulo, 1993–2016.

Characteristics		Males			Females			Total		
		N°	%	CMR	N°	%	CMR	N°	%	CMR
Causes										
	All causes	642	100.0	698.6	139	100.0	431.2	781	100.0	629.2
	All cancers	139	21.7	151.3	57	41.0	176.8	196	25.1	157.9
	All causes except cancer	503	78.3	547.4	82	59.0	254.4	585	74.9	471.3
Organic system										
	Digestive	64	46.0	69.6	16	28.1	49.6	80	40.8	64.4
	Respiratory	32	23.0	34.8	10	17.5	31.0	42	21.4	33.8
	Genitourinary	15	10.8	16.3	21	36.8	65.1	36	18.4	29.0
	Hematogenous	13	9.4	14.1	5	8.8	15.5	18	9.2	14.5
	Musculoskeletal	3	2.2	3.3	2	3.5	6.2	5	2.6	4.0
	Eyes and Central Nervous System	6	4.3	6.5	2	3.5	6.2	8	4.1	6.4
	Skin	1	0.7	1.1	1	1.8	3.1	2	1.0	1.6
	Uncertain	5	3.6	5.4	0	0.0	0.0	5	2.6	4.0
Total		139	100.0	151.3	57	100.0	176.8	196	100.0	157.9

CMR: crude mortality rate/100,000 person-year.

Table 3 shows SMR in external analysis. A lower risk of deaths from all causes was observed in the study population with SMR=0.224 (95%CI 0.208–0.240) when compared to the population of the city of São Paulo, as well as between men and women.

**Table 3:** Mortality by cancer – external comparison*: Number of observed and expected deaths, standardized mortality ratio, and 95% confidence interval, according to gender and cause of death; company working in research, development, and applications in the radiological and nuclear areas, São Paulo, 1996–2016.

			Males				Females				Total			
			O	E	SMR	95%CI	O	E	SMR	95%CI	O	E	SMR	95%CI
Causes of death														
	All causes		623	2,717.5	0.229	0.462–0.541	137	677.6	0.202	0.170–0.239	760	3,395.0	0.224	0.208–0.240
	All cancers		137	352.0	0.389	0.327–0.460	56	88.0	0.636	0.481–0.826	193	440.0	0.439	0.379–0.505
	All causes except cancer		486	2,365.4	0.205	0.188–0.225	81	589.6	0.137	0.109–0.171	567	2,955.0	0.192	0.176–0.208
Cancer groups														
Type														
	Solid		119	308.3	0.386	0.320–0.462	51	75.8	0.673	0.501–0.884	170	384.1	0.443	0.379–0.514
		Unspecified	5	18.5	0.270	0.087–0.629	0	5.2	0.000	---. 0.701	5	23.8	0.210	0.068–0.491
		Hematogenous	13	24.9	0.523	0.278–0.894	5	6.9	0.720	0.232–1.679	18	31.8	0.566	0.335–0.895
	Risk factor													
		Related to alcoholism	51	138.5	0.368	0.274–0.484	29	37.2	0.779	0.521–1.118	80	175.7	0.455	0.361–0.567
		Related to smoking	96	234.6	0.409	0.331–0.500	26	43.0	0.605	0.395–0.886	122	277.6	0.439	0.365–0.525
		Related to work	94	232.4	0.405	0.327–0.495	48	61.5	0.780	0.575–1.035	142	293.9	0.483	0.537–0.647
		Gamma and X radiation	77	191.0	0.403	0.318–0.504	45	51.5	0.873	0.637–1.168	122	242.5	0.503	0.418–0.601
	Organic system													
		Digestive	63	149.7	0.421	0.323–0.538	15	30.2	0.497	0.278–0.820	78	179.9	0.434	0.343–0.541
		Respiratory	32	73.8	0.434	0.297–0.612	10	9.8	1.017	0.487–1.871	42	83.6	0.502	0.362–0.679
		Genitourinary	15	62.1	0.242	0.135–0.399	21	29.5	0.712	0.440–1.088	36	91.6	0.393	0.275–0.544
		Musculoskeletal	3	4.3	0.691	0.139–2.020	2	1.3	1.516	0.170–5.470	5	5.7	0.883	0.285–2.062
		Skin	1	5.8	0.173	0.002–0.961	1	1.2	0.850	0.011–4.715	2	7.0	0.287	0.032–1.036
		Eyes and Central Nervous System	5	11.6	0.432	0.139–1.008	2	3.3	0.608	0.068–2.195	7	14.9	0.471	0.189–0.971
		Endocrine	0	1.5	0.000	---. 2.397	0	0.6	0.000	---. 5.731	0	2.2	0.000	---. 1.690
		Hematopoietic	13	26.0	0.501	0.266–0.856	5	7.2	0.693	0.223–1.616	18	33.2	0.543	0.321–0.857
		Undetermined	5	17.0	0.295	0.095–0.687	0	4.8	0.000	---. 0.761	5	21.8	0.229	0.074–0.535

O: observed deaths; E: expected deaths; SMR: standardized mortality ratio; 95%CI: 95% confidence interval.

* Standardization by gender, age group and calendar period, using the indirect standardization method, taking as the standard population the population of the Municipality of São Paulo in the respective calendar period.

External analysis showed a lower risk of death from all cancers in the study population, with SMR=0.439 (95%CI 0.379–0.505) when compared to the population of the city of São Paulo, a pattern also observed among men and women.

Similar results were observed in relation to deaths by types of cancer (solid, unspecified, and hematogenous), by risk factor (alcoholism, smoking, work, and gamma and X radiation), and between the different organic systems. Exceptions occurred in females, showing no statistically significant difference in relation to the city of São Paulo in terms of deaths from hematogenous cancers, cancers related to alcoholism, work, gamma and X radiation, and almost all organic systems.

Table 4 shows SMR in the internal analysis. A lower risk of deaths from all causes was observed in the monitored subgroup with SMR=0.685 (95%CI 0.618–0.758) when compared to the unmonitored subgroup, as well as between men and women.

**Table 4:** Cancer mortality – internal comparison*: Number of observed and expected deaths, standardized mortality ratio, and 95% confidence interval, according to gender and cause of death; company working in research, development, and applications in the radiological and nuclear areas, São Paulo, 1993–2016.

			Males				Females				Total			
			O	E	SMR	95%CI	O	E	SMR	95%CI	O	E	SMR	95%CI
Causes of death														
	All causes		316	470.6	0.671	0.599–0.750	62	81.3	0.763	0.585–0.978	378	551.9	0.685	0.618–0.758
	All cancers		72	95.3	0.756	0.591–0.952	23	36.1	0.637	0.404–0.956	95	131.4	0.723	0.585–0.884
	All causes except cancer		244	375.4	0.650	0.571–0.737	39	45.2	0.863	0.613–1.179	283	420.6	0.673	0.597–0.756
Cancer groups														
	Type													
		Solid	62	83.7	0.741	0.568–0.949	20	34.2	0.586	0.357–0.904	82	117.9	0.696	0.553–0.864
		Unspecified	3	0.0	---	---. ---	0	2.2	0.000	---. 1.645	3	2.2	1.344	0.270–3.931
		Hematogenous	7	8.5	0.826	0.331–1.703	3	1.9	1.555	0.312–4.542	10	10.4	0.961	0.460–1.768
	Risk factor													
		Related to alcoholism	29	32.8	0.885	0.592–1.271	13	18.3	0.709	0.377–1.213	42	51.1	0.822	0.592–1.111
		Related to smoking	49	68.2	0.719	0.532–0.951	8	20.2	0.395	0.170–0.779	57	88.4	0.645	0.488–0.836
		Related to work	53	61.8	0.858	0.642–1.122	20	31.1	0.643	0.393–0.993	73	92.9	0.786	0.616–0.988
		Gamma and X radiation	44	50.5	0.871	0.633–1.169	19	29.2	0.651	0.392–1.017	63	79.7	0.790	0.607–1.011
	Organic system													
		Digestive	36	39.4	0.913	0.639–1.264	6	10.6	0.564	0.206–1.227	42	50.1	0.839	0.604–1.134
		Respiratory	12	28.7	0.418	0.216–.730	3	7.5	0.399	0.080–1.167	15	36.2	0.414	0.232–0.683
		Genitourinary	8	9.9	0.804	0.346–1.584	8	13.8	0.581	0.250–1.145	16	23.7	0.674	0.385–1.095
		Musculoskeletal	2	1.3	1.589	0.178–5.731	2	0.0	---	---. ---	4	1.3	3.177	0.854–8.128
		Skin	0	1.3	0.000	---. 2.911	1	0.0	---	---. ---	1	1.3	0.794	0.010–4.416
		Eyes and Central Nervous System	4	3.1	1.296	0.348–3.314	0	2.2	0.000	---. 1.645	4	5.3	0.752	0.202–1.925
		Endocrine	0	0.0	---	---. ---	0	0.0	---	---. ---	0	0.0	---	---. ---
		Hematopoietic	7	8.5	0.826	0.331–1.703	3	1.9	1.555	0.312–4.542	10	10.4	0.961	0.460. –1.768
		Undetermined	3	3.1	0.972	0.195–2.837	0	0.0	---	---. ---	3	3.1	0.972	0.195–2.837

O: observed deaths; E: expected deaths; SMR: standardized mortality ratio; 95%CI: 95% confidence interval.

* Standardization by gender, age range, and calendar period, using the indirect standardization method, taking as the standard population the study population not monitored for gamma and X radiation in the respective calendar period.

Internal analysis showed a lower risk of death from all cancers in the monitored subgroup with SMR=0.723 (95%CI 0.585–0.884) when compared to the unmonitored subgroup, in males and females.

The results observed showed that deaths by types of cancer (solid, unspecified, and hematogenous), by risk factor (alcoholism, smoking, work, and gamma and X radiation), and between different organic systems did not present statistically significant differences between the monitored and unmonitored subgroups. Exceptions occurred among solid cancers, those related to smoking and the respiratory system, which showed a lower risk of death among the ones followed-up.

## DISCUSSION

This work sought to compare cancer mortality among workers at a company in the radiological and nuclear areas in the city of São Paulo with the general population of workers followed-up and not followed-up for gamma and X radiation.

The main causes of death were cardiovascular diseases, neoplasms and ill-defined causes, a profile similar to that currently observed worldwide^23^ and in Brazil^24^, although, in some countries, cancers are the first cause of death in the age range up to 69 years old^25^. Ill-defined causes were possibly due to limited quality of available data, including potential under-registration of cancer deaths in the state of São Paulo and coverage of death registration periods, initiated by PRO-AIM in the city of São Paulo in 1996, with no records of deaths among workers prior to that period and in other locations.

Standardized mortality ratios in external analysis showed that the risks of death from all causes were lower in the study population, in males and females, compared to those in the city of São Paulo. These results suggest the healthy worker effect. In relation to other studies^10,11,13,26–29^, SMR were not as low as those observed in this study. This points to the contribution that limited information on deaths may have had on this result as well as to the positive effects of preventive strategies applied to the study population.

The risk of deaths from all cancers showed a less pronounced reduction than the risk of deaths from all causes (excluding cancers), when compared with the population of the city of São Paulo. This was observed in the study population and both in male and female strata. This difference between standardized mortality ratios (from all causes except cancer and from cancer) is consistent with the healthy worker effect, which is generally less marked for cancer deaths than for other causes of death^13,14,30,31^.

Among the factors that may have contributed to the healthy worker effect were access to health services by the study population, both through medical insurance and occupational health services, promoting treatment of diseases, examinations to detect risk factors of cardiovascular diseases and cancer screening, leading to a reduction in mortality^31^. The higher proportion of cancer deaths in the highest socioeconomic level shows that access to education and medical care may have led to an increase in cancer diagnoses.

Occupational exposure in the external analysis was assessed indirectly through the selection of cancers related to occupational exposures with sufficient evidence in humans^20^. Even without the selection of cancers related only to chemical substances, this allowed the indirect assessment of exposure to chemicals, for which no systematic records were found in the company. Both men and women in the study population had a lower risk of mortality from cancers associated with occupational exposure. A similar result was obtained through the analysis of the group of radiogenic cancers with sufficient evidence in humans^20^, which showed a lower risk of death due to exposure to gamma and X radiation in the study population, among men and women, which was also observed in other studies^3^. These results can again be attributed to preventive strategies and access to health care; however, they do not rule out the possibility that individually considered cases may be linked to exposure to carcinogenic agents present in the work environment.

Internal mortality analysis evaluated the monitored subgroup against the non-monitored one using the latter as a reference. Internal analysis had the advantage of comparing more similar populations in relation to factors linked to the healthy worker effect. Results showed that the monitored subgroup, of men and women, had a lower risk of mortality from all causes compared to the unmonitored one. This result suggests an internal healthy worker effect^3,32,33^. In relation to cancer mortality, there was a lower risk of cancer deaths in the monitored subgroup and between the male and female strata. Similar results were found in the literature^2,3,32^.

Lower SMR was more marked in mortality from all causes than from all cancers among monitored men and in the monitored subgroup, but among monitored women, this reduction was more present in mortality from all cancers. This result may be a random event due to the small number of deaths among women, or point to the possibility that women resorted to screening and detection of cancers. Breast cancer can be detected in the early stages through mammography and cervical cancer through oncotic cytology, both screening methods that are already well established in public health^34–36^.

Results showed that there were no differences between the two groups in relation to deaths by types of cancer (solid, unspecified, and hematogenous), by risk factors (alcoholism, smoking, work, and gamma and X radiation), and between the different organic systems. Exceptions were solid cancers, those related to smoking and those of the respiratory system. These results suggest that there are possibly fewer smokers among monitored workers. Literature studies show that smoking is unlikely to be a risk factor for cancer among workers in the nuclear industry^37,38^. Other studies have shown similar results^39,40^.

The only organic system that showed a statistically significant increase in the risk of death in the monitored subgroup was the musculoskeletal system. However, due to the small number of deaths, it may have been a random event. Similar results have been reported in the literature^5,40–42^.

### Successes and limitations

The main limitations of the study regarded access and quality of data. Death records were restricted to the state of São Paulo; however, this is the state with the best socioeconomic level and mortality records. The relatively small number of deaths, especially among women, determined the option of grouping cancers in order to obtain more consistent results.

Flaws in dosimetric records, especially in the initial phases of the company, were also a limitation. Furthermore, 70% of the monitored population had an accumulated radiation dose of up to 10 mSv. This characteristic, positive from the worker's health point of view, was an impediment to an assessment by accumulated dose stratum, determining the categorization between monitored and unmonitored.

Despite the relatively small number of deaths, the option for standardized mortality ratio analysis provided consistent results, evaluating monitored and unmonitored workers for gamma and X radiation.

As a success, the 60-year follow-up of a cohort of workers is highlighted, a desirable period to analyze cancer mortality, which was a great benefit. The study also allowed to analyze a population with the majority of men and women over 50 years of age, a desirable age range for analyzing cancer mortality.

Although international cohorts of nuclear industry workers have had a larger number of participants^3–5,7,8,11,12,27,28,42^, this cohort had a relevant number and took place in one of the largest companies in this field in Brazil, making it possible to add knowledge to the national reality. Furthermore, this is one of the few evaluations of cancer deaths in females, which is uncommon among studies in the nuclear sector.

A prominent factor is the healthy worker effect, indicating that disease prevention and health promotion measures provided by occupational health, together with access to health services, reduced mortality from cancer and other diseases.

SMR showed lower mortality from cancer and all causes among exposed workers and among those monitored for gamma and X radiation, which can be attributed to the quality of protective measures in the workplace and health control of these workers. These results suggest the occurrence of the healthy worker effect and the benefits arising from good practices in health and safety at work.
