# Supplementary material for: Cancer mortality in workers at risk of occupational exposure to ionizing radiation in a company in the nuclear sector headquarters in São Paulo
Source: Rev Bras Epidemiol. 2024 Mar 18;27:e240011. doi: 10.1590/1980-549720240011 (PMC10946289; doi:10.1590/1980-549720240011)
Supplement: Supplementary file 1 [file 1980-5497-rbepid-27-e240011-Suppl01.docx]

**Material Suplementar**

Método Indireto de Padronização

No método indireto de padronização se comparam duas populações, sendo que uma é a referência. É muito utilizada para comparação dos dados de mortalidade em populações ocupacionalmente expostas.

O cálculo da razão de mortalidade padronizada (RMP) consiste na divisão da somatória dos números de óbitos observados de cada estrato etário da população de estudo pela somatória de óbitos esperados de cada estrato etário da mesma população. Os óbitos esperados são obtidos pelos produtos dos coeficientes de mortalidade específicos por idade da população de referência pelo número de pessoas-tempo da população de estudo, como mostra a equação abaixo^21^, sendo *i* o estrato etário:

$$RMP= \frac{\Sigma_{i}a_{i}}{\Sigma_{i}N_{1,i}(\frac{b_{i}}{N_{0i}})}$$

Onde:

ai = número de óbitos observados da população de estudo

bi = número de óbitos da população de referência

N_0_ = população de referência

N_1_ = número de pessoas tempo da população de estudo

Na análise externa para todo o período de análise, a RMP consistiu na razão da somatória dos óbitos observados de cada estrato etário de cada período calendário pela somatória dos óbitos esperados de cada estrato etário de cada período calendário da população de estudo para ambos os sexos separados e juntos, seguindo a seguinte fórmula:

$${RMP}_{n}^{k}=\frac{Ʃ_{i,j}^{k,n}oij}{Ʃ_{i,j}^{k,n}[\left( \frac{OIJ}{PIJ} \right)*pij]}$$

Onde:

oij = óbitos observados do estrato etário i do período calendário j da população de estudo

pij = população do estrato etário i do período calendário j da população de estudo (número de pessoas-ano)

OIJ= número de óbitos do estrato etário I do período calendário J da população padrão

PIJ= população do estrato etário I do período calendário J da população padrão

i= número do estrato etário

j= número do estrato do período calendário

k = número de estratos etários

n= número de estratos de período calendário

Foi estimado o intervalo de confiança de 95 % da RMP e foi aceita significância estatística se p<0,05.

O cálculo da RMP na análise interna comparou o número de óbitos observados no subgrupo monitorado para radiação gama e X da população de estudo com o número de óbitos esperados após padronização pelo subgrupo não monitorado da população de estudo. Foi estimado o intervalo de confiança de 95 % da RMP e foi aceita significância estatística se p<0,05.

O numerador da razão de mortalidade padronizada por idade foi composta pela somatória do número de óbitos dos estratos etários dos períodos calendários do subgrupo monitorado que compôs o número de óbitos observados. O denominador da RMP por idade foi obtida pela somatória dos resultados dos produtos dos coeficientes de mortalidade específicos por idade de cada estrato etário de cada período calendário do subgrupo não monitorado pelo número de pessoas-ano dos mesmos estratos etários e dos mesmos períodos calendários do subgrupo monitorado. O valor resultante desta somatória compôs o número de óbitos esperados. A RMP por idade por período calendário resultou da razão entre o número de óbitos observados do subgrupo monitorado pelo número de óbitos esperados para cada estrato etário de cada período calendário. Assim, a RMP da análise interna foi calculada para ambos os sexos separados e juntos a partir da seguinte fórmula:

$${RMP}_{n}^{k}=\frac{Ʃ_{i,j}^{k,n}oij}{Ʃ_{i,j}^{k,n}[\left( \frac{OIJ}{PIJ} \right)*pij]}$$

Onde:

oij = óbitos observados do estrato etário i do período calendário j do subgrupo monitorado

pi = população do estrato etário i do período calendário j do subgrupo monitorado

OI= óbitos observados do estrato etário i do período calendário J do subgrupo não monitorado

PIJ= população do estrato etário i do período calendário J do subgrupo não monitorado

i=número do estrato etário

j= número do estrato do período calendário

k = número de estratos etários

n= número de estratos de período calendário

**Quadro A: Agrupamento dos cânceres (códigos da CID 10) segundo o fator de risco e o sistema orgânico**

| **Fator de risco** | **Cap. C da CID-10** | **Cap. D da CID-10** |
| --- | --- | --- |
| Etilismo | C00 - C06 | D00 |
|  | C09 - C10 |  |
|  | C12 - C14 |  |
|  | C15 |  |
|  | C16 |  |
|  | C18 - C21 |  |
|  | C22 |  |
|  | C32 |  |
|  | C50 |  |
| Tabagismo | C00 - C06 | D00 |
|  | C09 - C14 |  |
|  | C15 |  |
|  | C16 |  |
|  | C18 - C21 |  |
|  | C22 |  |
|  | C25 |  |
|  | C32 |  |
|  | C33 - C34 |  |
|  | C53 | D06 |
|  | C64 - C66 |  |
|  | C67 |  |
|  | C92 |  |
| Ocupacional | C07 - C08 |  |
|  | C11 |  |
|  | C15 |  |
|  | C16 |  |
|  | C18 - C21 |  |
|  | C22 |  |
|  | C30 |  |
|  | C31 |  |
|  | C32 |  |
|  | C33 - C34 |  |
|  | C40 - C41 |  |
|  | C43 - C44 | D03 - D04 |
|  | C45 |  |
|  | C50 | D05 |
|  | C56 |  |
|  | C64 |  |
|  | C67 |  |
|  | C69 |  |
|  | C70 - C72 | D32 - D33 |
|  |  | D42 - D43 |
|  | C73 |  |
|  | C82 - C85 |  |
|  | C91 - C95 |  |
| Radiação gama e X | C07 - C08 |  |
|  | C15 |  |
|  | C16 |  |
|  | C18 - C21 |  |
|  | C30 |  |
|  | C31 |  |
|  | C33 - C34 |  |
|  | C40 - C41 |  |
|  | C43 - C44 | D03 - D04 |
|  | C50 | D05 |
|  | C64 |  |
|  | C67 |  |
|  | C70 - C72 | D32 - D33 |
|  |  | D42 - D43 |
|  | C73 |  |
|  | C91 - C95 |  |
| **Sistema orgânico** | **Cap. C da CID-10** | **Cap. D da CID-10** |
| Trato digestório | C00 - C08 | D00 - D01 |
|  | C10 | D37 |
|  | C13 - C26 |  |
| Trato respiratório e órgãos torácicos | C09 | D02 |
|  | C11 - C12 | D38 |
|  | C31 - C39 |  |
| Ossos e tecidos moles | C40 - C41 |  |
|  | C45 - C49 |  |
| Pele | C43 - C44 | D03 -D04 |
| Trato geniturinário | C50 - C68 | D05 - D07 |
|  |  | D39 - D41 |
| Olhos e Sistema nervoso central | C69 - C70 - C72 | D32 - D33 |
|  |  | D42 - D43 |
| Sistema endócrino | C73 - C75 | D44 |
| Sítio indeterminado | C76 - C80 | D09 |
|  | C97 | D48 |
| Sistema hematopoiético | C81 - C96 | D45 - D47 |

**Tabela A**: Óbitos por sexo segundo capítulo da CID 10, Empresa com trabalho em pesquisa, desenvolvimento e aplicações nas áreas Radiológica e Nuclear, São Paulo, 1956 – 2016.

| **Causa do óbito** | **CID 10** | **Masculino** | | **Feminino** | | **Total** | |
| --- | --- | --- | --- | --- | --- | --- | --- |
|  |  | **N** | **%** | **N** | **%** | **N** | **%** |
| Distúrbios cardiovasculares | I | 198 | 28,5 | 32 | 21,8 | 230 | 27,3 |
| Neoplasias | C+D^1^ | 139 | 20 | 57 | 38,8 | 196 | 23,3 |
| Causas mal definidas | R | 143 | 20,6 | 25 | 17 | 168 | 20 |
| Doenças respiratórias | J | 49 | 7,1 | 6 | 4,1 | 55 | 6,5 |
| Acidentes | V+W+X+Y | 45 | 6,5 | 5 | 3,4 | 50 | 5,9 |
| Distúrbios gastrointestinais | K | 40 | 5,8 | 6 | 4,1 | 46 | 5,5 |
| Doenças infecciosas e parasitárias | A+B | 29 | 4,2 | 5 | 3,4 | 34 | 4 |
| Distúrbios do metabolismo e nutrição | E | 29 | 4,2 | 3 | 2 | 32 | 3,8 |
| Doenças geniturinárias | N | 13 | 1,9 | 4 | 2,7 | 17 | 2 |
| Distúrbios neurológicos | G | 4 | 0,6 | 2 | 1,4 | 6 | 0,7 |
| Doenças do sistema hematopoiético | D68+D69 | 2 | 0,3 | 1 | 0,7 | 3 | 0,4 |
| Distúrbios osteomusculares | M | 2 | 0,3 | 0 | 0 | 2 | 0,2 |
| Transtornos mentais | F | 1 | 0,1 | 0 | 0 | 1 | 0,1 |
| Doenças da pele | L | 0 | 0 | 1 | 0,7 | 1 | 0,1 |
| Traumas | T | 1 | 0,1 | 0 | 0 | 1 | 0,1 |
| **Total** |  | 695 | 100 | 147 | 100 | 842 | 100 |

^1^: lesões precursoras das neoplasias malignas (D00 – D09), tumores cerebrais benignos (D32 e D33) e neoplasias de comportamento incerto e hematopoiéticas (D37 – D48).

**Tabela B**: Óbitos por sítio de câncer por sexo, Empresa com trabalho em pesquisa, desenvolvimento e aplicações nas áreas Radiológica e Nuclear, São Paulo, 1956 – 2016

| **Sítio do câncer** | **CID 10** | **Masculino** | | **Feminino** | | **Total** | |
| --- | --- | --- | --- | --- | --- | --- | --- |
|  |  | **N** | **%** | **N** | **%** | **N** | **%** |
| Boca NE | C06 | 1 | 0,7 | 0 | 0 | 1 | 0,5 |
| Orofaringe | C10 | 1 | 0,7 | 0 | 0 | 1 | 0,5 |
| Cavidade oral | C14 | 1 | 0,7 | 0 | 0 | 1 | 0,5 |
| Esôfago | C15 | 7 | 5 | 0 | 0 | 7 | 3,6 |
| Estômago | C16 | 13 | 9,4 | 4 | 7 | 17 | 8,7 |
| Intestino delgado | C17 | 1 | 0,7 | 0 | 0 | 1 | 0,5 |
| Intestino grosso | C18-C21 | 16 | 11,5 | 8 | 14 | 24 | 12,2 |
| Fígado | C22 | 8 | 5,8 | 0 | 0 | 8 | 4,1 |
| Vias biliares | C24 | 2 | 1,4 | 1 | 1,8 | 3 | 1,5 |
| Pâncreas | C25 | 12 | 8,6 | 3 | 5,3 | 15 | 7,7 |
| Neoplasia trato digestório | C26 | 2 | 1,4 | 0 | 0 | 2 | 1 |
| Laringe | C32 | 5 | 3,6 | 0 | 0 | 5 | 2,6 |
| Brônquios e pulmões | C34 | 27 | 19,4 | 10 | 17,5 | 37 | 18,9 |
| Ossos | C41 | 3 | 2,2 | 2 | 3,5 | 5 | 2,6 |
| Pele melanoma | C43 | 1 | 0,7 | 1 | 1,8 | 2 | 1 |
| Mama | C50 | 0 | 0 | 18 | 31,6 | 18 | 9,2 |
| Colo do útero | C53 | 0 | 0 | 1 | 1,8 | 1 | 0,5 |
| Útero SOE | C55 | 0 | 0 | 1 | 1,8 | 1 | 0,5 |
| Próstata | C61 | 11 | 7,9 | 0 | 0 | 11 | 5,6 |
| Rim | C64 | 2 | 1,4 | 1 | 1,8 | 3 | 1,5 |
| Bexiga | C67 | 2 | 1,4 | 0 | 0 | 2 | 1 |
| Sistema nervoso central | C71 | 4 | 2,9 | 2 | 3,5 | 6 | 3,1 |
| Neoplasias local mal definido | C76 | 2 | 1,4 | 0 | 0 | 2 | 1 |
| Neopl malig secund org respirat e digestivos | C78 | 1 | 0,7 | 0 | 0 | 1 | 0,5 |
| Neoplasia maligna sem localização | C80 | 2 | 1,4 | 0 | 0 | 2 | 1 |
| Linfoma de Hodgkin | C81 | 1 | 0,7 | 1 | 1,8 | 2 | 1 |
| Linfoma de Burkitt | C83 | 1 | 0,7 | 0 | 0 | 1 | 0,5 |
| Linfoma não Hodgkin | C85 | 2 | 1,4 | 2 | 3,5 | 4 | 2 |
| Mieloma múltiplo | C90 | 3 | 2,2 | 1 | 1,8 | 4 | 2 |
| Leucemia linfóide crônica | C91 | 0 | 0 | 1 | 1,8 | 1 | 0,5 |
| Leucemia mielóide aguda | C92 | 2 | 1,4 | 0 | 0 | 2 | 1 |
| Leucemia SOE | C95 | 1 | 0,7 | 0 | 0 | 1 | 0,5 |
| Meningioma | D32 | 2 | 1,4 | 0 | 0 | 2 | 1 |
| Síndrome mielodisplásica | D46 | 1 | 0,7 | 0 | 0 | 1 | 0,5 |
| Outras neoplasias do tecido linfático e hematopoiético | D47 | 2 | 1,4 | 0 | 0 | 2 | 1 |
| **Total** |  | 139 | 100 | 57 | 100 | 196 | 100 |
